# Lubrication for Osteoarthritis: From Single-Function to Multifunctional Lubricants

**DOI:** 10.3390/ijms26051856

**Published:** 2025-02-21

**Authors:** Wen Chen, Qianwen Ye, Mingshuo Zhang, Renjian Xie, Chunming Xu

**Affiliations:** 1 School of Rehabilitation Medicine, Gannan Medical University, Ganzhou 341000, China; chenwen61@gmu.cn; 2 Jiangxi Provincial Key Laboratory of Tissue Engineering (2024SSY06291), Gannan Medical University, Ganzhou 341000, China; yeqianwen@gmu.edu.cn (Q.Y.); zhangmingshuo@gmu.edu.cn (M.Z.); 3 School of Medical Information Engineering, Gannan Medical University, Ganzhou 341000, China; 4 Key Laboratory of Prevention and Treatment of Cardiovascular and Cerebrovascular Diseases (Ministry of Education), Gannan Medical University, Ganzhou 341000, China; 5 School of Basic Medicine, Gannan Medical University, Ganzhou 341000, China

**Keywords:** articular cartilage, biolubrication, osteoarthritis, hydrogels, stem cells, nanoparticles, anti-inflammatory drugs

## Abstract

Osteoarthritis (OA) is a common degenerative joint disease that progressively destroys articular cartilage, leading to increased joint friction and severe pain. Therefore, OA can be treated by restoring the lubricating properties of cartilage. In this study, recent advances in lubricants for the treatment of OA are reviewed for both single-function and multifunctional lubricants. Single-function lubricants mainly include glycosaminoglycans, lubricin, and phospholipids, whereas multifunctional lubricants are composed of lubricating and anti-inflammatory bifunctional hydrogels, stem cell-loaded lubricating hydrogels, and drug-loaded lubricating nanoparticles. This review emphasizes the importance of restoring joint lubrication capacity for the treatment of OA and explores the structural features, lubrication properties, and role of these lubricants in modulating intracellular inflammatory responses and metabolism. Current challenges and future research directions in this field are also discussed, with the aim of providing a scientific basis and new ideas for the clinical treatment of OA.

## 1. Introduction

Articular cartilage serves as cushioning tissue at the ends of synovial joints, particularly in the knee, functioning as a highly efficient water-based friction system. With a friction coefficient as low as 10^−3^ and the ability to withstand pressures up to 18 MPa [1,2], it ensures frictionless and painless joint movement, supporting daily activity and mobility [3,4]. Current research attributes the superior lubricating performance of articular cartilage to the lubricating molecules in synovial fluid, primarily hyaluronic acid (HA), lubricin, and phospholipids [5,6,7,8,9]. These molecules work synergistically to form a brush-like structure with HA as the backbone, lubricin as the side chains, and phospholipids anchored to the HA due to their high affinity [10,11,12]. Healthy articular cartilage is a self-lubricating system that actively maintains lubrication between cartilage surfaces throughout its lifecycle [13].

Osteoarthritis (OA) is a multifactorial joint disease characterized by irreversible structural and functional changes in articular cartilage. It affects over 70% of individuals aged 55 to 77 [14,15,16]. Notably, the first structural changes occur in the outer surface of articular cartilage, leading to a coefficient of friction (COF) much higher than what is observed in healthy joints [17]. Increased friction triggers local inflammation of the synovium. This leads to the overproduction of cytokines (e.g., interleukins, tumor necrosis factor-α) and enzymes (e.g., matrix metalloproteinases). These factors promote the degradation of articular cartilage, driving the progression of OA from initiation to the early and, ultimately, end stages [18,19]. Therefore, the role of lubrication in the initiation and development of OA has attracted attention, and various lubricants have been designed to treat OA [1,20,21,22,23].

**Figure 1:**
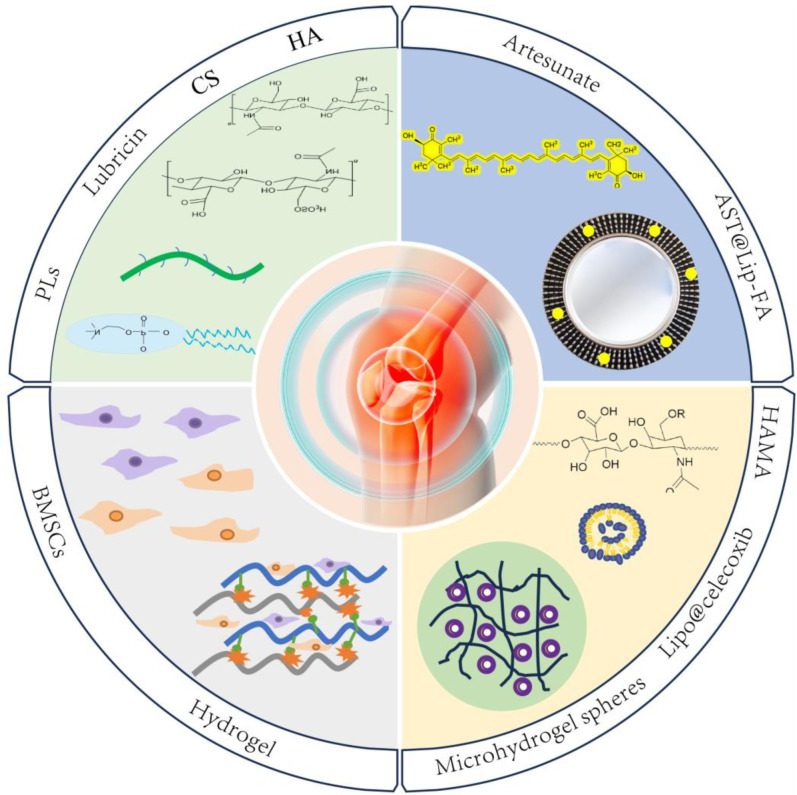
Lubrication for OA treatment, ranging from single-function lubricants to multifunctional lubricant strategies. Single-function lubricants include glycosaminoglycans (e.g., chondroitin sulfate (CS) and hyaluronic acid (HA)), lubricin, and phospholipids. Multifunctional lubricants include lubricating and anti-inflammatory hydrogels, stem cell-loaded lubricants (e.g., bone mesenchymal stem cells (BMSCs)), and drug-loaded nanoparticles (e.g., celecoxib or artesunate (AST) in liposomes (Lipo@celecoxib and AST@lipo, respectively)).

In this review, we first summarize the status of lubricants, addressing both single-function lubricants and multifunctional lubricants, and their use in alleviating symptoms of osteoarthritis (OA) [24,25]. Single-function lubricants primarily include glycosaminoglycans (GAGs), lubricin, and lipids in the joints and their derivatives [26]. These lubricants primarily reduce joint friction and provide temporary relief by mimicking the natural lubricants present in healthy joints. Key examples of single-function lubricants are hyaluronic acid (HA), chondroitin sulfate, and phospholipids. While they are effective in reducing friction, they do not address the underlying inflammation or tissue degeneration present in OA [27,28,29], In contrast, multifunctional lubricants incorporate additional therapeutic properties beyond friction reduction [30]. These lubricants are designed to address both the mechanical and biological factors of OA [31,32]. They combine lubrication with anti-inflammatory effects and tissue regeneration properties. Examples include lubricating and anti-inflammatory bifunctional hydrogels, stem cell-loaded lubricating hydrogels, and drug-loaded lubricating nanoparticles (as illustrated in Figure 1) [30,33,34,35]. Multifunctional lubricants are designed to provide more comprehensive treatment, targeting multiple pathways involved in OA progression, including inflammation, cartilage degradation, and tissue regeneration [36]. Finally, the current limitations and future perspectives of both types of lubricants are discussed, providing a scientific basis and new ideas for the clinical treatment of OA and emphasizing the need for more effective and long-lasting therapeutic solutions.

## 2. Single-Function Lubricants

Single-function lubricants consist mainly of glycosaminoglycans (GAGs), which are lubricating proteins and lipids in the joints [37,38]. These constituents play an essential role in joint lubrication, helping to reduce joint friction and protect cartilage [1]. Some GAGs, such as HA and chondroitin sulfate, comprise an important class with a wide range of applications in medicine due to their anti-inflammatory and immunomodulatory activities [39]. Lubricating proteins, such as lubricin, primarily exist in synovial fluid and are essential for joint lubrication and protecting cartilage [32]. Phospholipids are another key component of joint lubrication as they help maintain lubricity and elasticity [28,29]. Table 1 summarizes the currently available friction data of single-function lubricants.

### 2.1. Hyaluronic Acid

HA, also known as vitreous acid, was first isolated from bovine vitreous fluid by Meter and Palmer in 1934 [54]. HA is an important component of the extracellular matrix [55]. It consists of repeating disaccharide units of N-acetylglucosamine and D-glucuronic acid, which has a highly negative charge and exhibits strong hydrophilicity [56]. HA has good viscoelasticity and strain properties with unique hydrodynamics [57], and is an important component of the cartilage matrix and synovial fluid, providing good lubrication for articular cartilage [58,59]. The molecular weight of HA in healthy synovial fluid ranges from 1 to 10.9 MDa [22,60] but decreases to 4.2 MDa in OA synovial fluid [61]. However, as the molecular weight of HA decreases, the viscosity of the synovial fluid decreases, and wear on the articular surface increases, eventually exacerbating the symptoms of OA [34].

**Figure 2:**
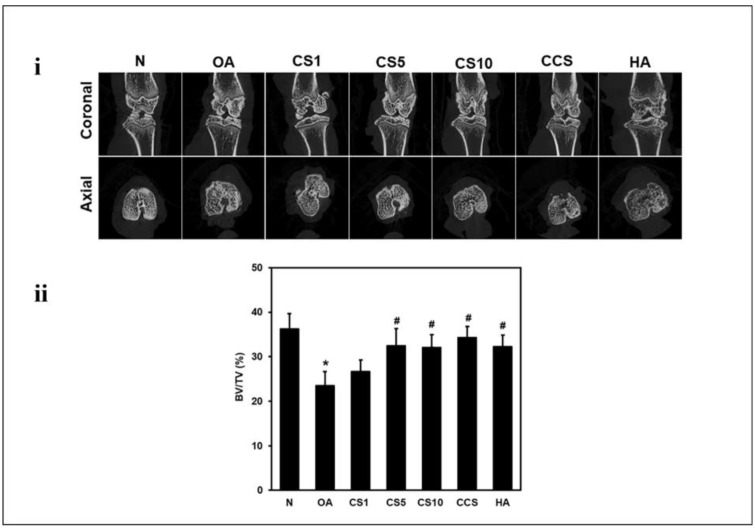
(**i**) Effects of HA on MNX-induced cartilage and bone injury in OA rats after 6 weeks of treatment. Micro-CT images and coronal sagittal and axial images were obtained via micro-CT; (**ii**) quantification BV of calcified meniscus and synovium. Data are expressed as mean ± SD (n = 6). The differences between treatments with different letters are statistically significant (*p* < 0.05). Reprinted with permission from Ref. [62], copyright 2024, Srinivasan et al. # and * indicate statistical significance (*p* < 0.05) when comparing with the control and blank groups, respectively.

In an animal experiment, Perisasamy et al. tested the efficacy of HA in an OA model induced via surgical meniscectomy (MNX). Their experimental results showed that HA rebuilt the injured cartilage and reduced the progression of OA (as shown in Figure 2) [62]. Hayes et al. studied high-molecular-weight HA (HMW-HA) and found it to be more favorable than low-molecular-weight HA (LMW-HA) because HMW-HA has a greater affinity for CD44 receptors. Therefore HMW-HA can more effectively inhibit the production of matrix metalloproteinase-1 (MMP-1) [60]. After HA-CD44 binding, the expression of adisintegrin and metalloproteinase with thrombospondin motifs (ADAMTS) was decreased [63]. The ADAMTS family participates in the cleavage of synovial components, resulting in reduced synovial cleavage and thereby increasing the viscosity of synovial fluid, reducing friction between joints and promoting recovery from OA [64,65,66]. Cathepsin K is a protease involved in both bone remodeling and resorption, as well as in the degradation of articular cartilage in OA. Mochihito’s research indicates that HMW-HA affects the efficacy of OA treatment by inhibiting cathepsin K expression through the suppression of NF-κB [67].

Clinical data show that HMW-HA is often injected into the joint to relieve mild OA symptoms in a procedure called viscosupplementation [60,68]. The 2019 and 2023 evidence-based medicine guidelines for knee osteoarthritis recommend the use of HA supplementation therapy for symptomatic OA patients [69,70]. Demirhan et al. investigated the effects of different doses of linear HMW-HA injections on patients with OA. The study was a randomized, single-blind trial that enrolled 100 patients and randomly assigned them to three groups receiving 20 mg, 32 mg, or 48 mg HA injections. Pain, stiffness, and function were assessed using the Visual Analog Scale (VAS) and the Western Ontario and McMaster Universities Osteoarthritis Index (WOMAC). Additionally, quality of life, overall patient assessment, and Timed Up and Go (TUG) test scores were evaluated. The results showed significant improvements in WOMAC, VAS, quality of life, overall patient assessment, and TUG scores at all follow-up time points (*p* < 0.001), demonstrating that the intra-articular injection of different doses of linear HMW-HA significantly improves quality of life in knee OA patients over the course of six months [71]. Tang et al. evaluated the long-term efficacy and safety of multiple HA injections for knee pain. Eighty-five patients with knee pain received two cycles of HA treatment over five weeks, with clinical assessments conducted twenty-four months after the first cycle. The primary efficacy endpoint was the VAS pain score, with secondary endpoints including WOMAC scores and overall assessments by patients and physicians. Safety was assessed based on adverse events (AEs). In total, 71 patients (83.5%) completed the final study. VAS scores significantly decreased from baseline (65.06 ± 12) to 30.17 ± 11.92 at 6 months and were maintained at 35.79 ± 7.92 at 24 months (*p* < 0.01). The secondary variables (WOMAC A, B, and C scores, total WOMAC score, and overall assessments by patients and physicians) supported these findings. The incidence of AEs during the first and second cycles was 9.4% and 8.2%, respectively, with no severe adverse events reported, demonstrating the efficacy of multiple HA injections for OA [72]. K D Brandt et al. conducted a prospective, multicenter, randomized, double-blind, controlled trial in 226 OA patients to evaluate the safety and efficacy of HA. Patients were randomly assigned to receive either 30 mg injections of HA three times a week or a control group that received saline, with follow-up lasting 25 weeks. Compared to the control group, the HA group showed significant improvement in their WOMAC pain scores, global assessments by patients and investigators, and pain while standing from week 7 to 27. Of the patients treated with HA, 58% achieved a 5-point or greater improvement in mean pain scores from week 7 to 27, compared to 40% in the control group. Additionally, nearly twice as many patients in the HA group (30% vs. 17%) achieved at least a 7-point net improvement. Few side effects occurred, with no significant differences between groups (HA: 9 cases (8%); saline: 11 cases (10%)). The incidence of injection site reactions was low (HA: 1.2%: saline: 1.5%). These results indicate that HA treatment is well tolerated and significantly improves symptoms in patients with mild to moderate OA [73].

In contrast, it has been reported that the lubrication ability of HA may not vary with its molecular weight [74]. Veronica et al. compared the effects of low-molecular-weight (LMW-HA) and high-molecular-weight (HMW-HA) hyaluronic acid in rabbit OA. According to the Osteoarthritis Research Society International (OARSI), LMW-HA treatment reduced OA severity in rabbits from grade 3.4 to 1.5. HMW-HA treatment reduced OA severity from grade 3.4 to 2.2. The LMW-HA and HMW-HA injections produced similar effects in rabbit OA [75]. After inducing OA in horses, Henrique et al. injected LMW-HA (40 kDa, 20 mg/mL) in one group and HMW-HA (1350 kDa, 20 mg/mL) in the other [76,77]. Joint assessment and synovial fluid analysis were performed at 8, 24, and 48 h. Regarding pain response, both groups showed lameness after 8 h. After 24 h, the LMW-HA group had a significant reduction in leukocyte count (baseline 200–400 cells/μL → 100,000–200,000 cells/μL). After 48 h, both groups showed a minimal increase in cartilage degradation biomarkers at 24 h, with levels returning almost to baseline at 48 h. This indicates that HMW-HA and LMW-HA both have cartilage- and joint-protective effects [76].

### 2.2. Chondroitin Sulfate (CS)

CS is one of the important components of synovial fluid, and the concentration of CS in normal osteoarticular joints is 0.05–0.15 mg/mL [78]. The synthesis rate of CS decreases in advanced OA [79]. In vitro experiments have shown that a loss of CS leads to deformation and elevated COF in cartilage tissue [41]. Uebelhart et al. treated knee OA patients with oral CS, resulting in improved OA on imaging tests. CS had good tolerability and no serious adverse effects [80]. Additionally, the efficacy of oral CS was compared to a placebo group. The results showed that oral CS improved joint mobility and relieved pain [81].

Healthy articular cartilage experiences nature’s most efficient lubrication, with a coefficient of friction as low as 0.001 [1,82]. A study investigated whether different oral doses of CS affected efficacy. Results showed that 800 mg/day of CS was as effective as 1200 mg/day. It has been speculated that the efficacy of CS treatment for OA may not be closely related to dosage [83]. Katta et al. demonstrated that increasing CS concentrations can reduce friction. The static friction coefficient of 10 mg/mL CS is 0.89, while the static friction coefficient of 50 mg/mL CS is 0.57 [84]. In summary, the oral administration of CS can alleviate symptoms of OA, but its efficacy may not relate to the dosage. In case of strong drug resistance, the therapeutic effect of oral CS requires further research. In vitro experiments show that the lubrication effect of CS depends on its concentration. In addition, modifying CS can enhance its performance, making it a promising OA treatment material.

### 2.3. Lubricin

Superficial zonulin protein (SZP), also known as lubricin or PRG4 [22], is the most abundant GAG in human synovial fluid and is found on cartilage surfaces [42]. It has a mucin-like region with O-linked oligosaccharides that reduce friction through repulsive forces [37], and boundary lubrication is mediated by O-linked β-Gal-GalNAc oligosaccharides [43]. Lubricin is a glycoprotein with a molecular weight of 345 kDa [85]. This molecule is synthesized by superficial chondrocytes and synoviocytes and is present in synovial fluid [86].

**Figure 3:**
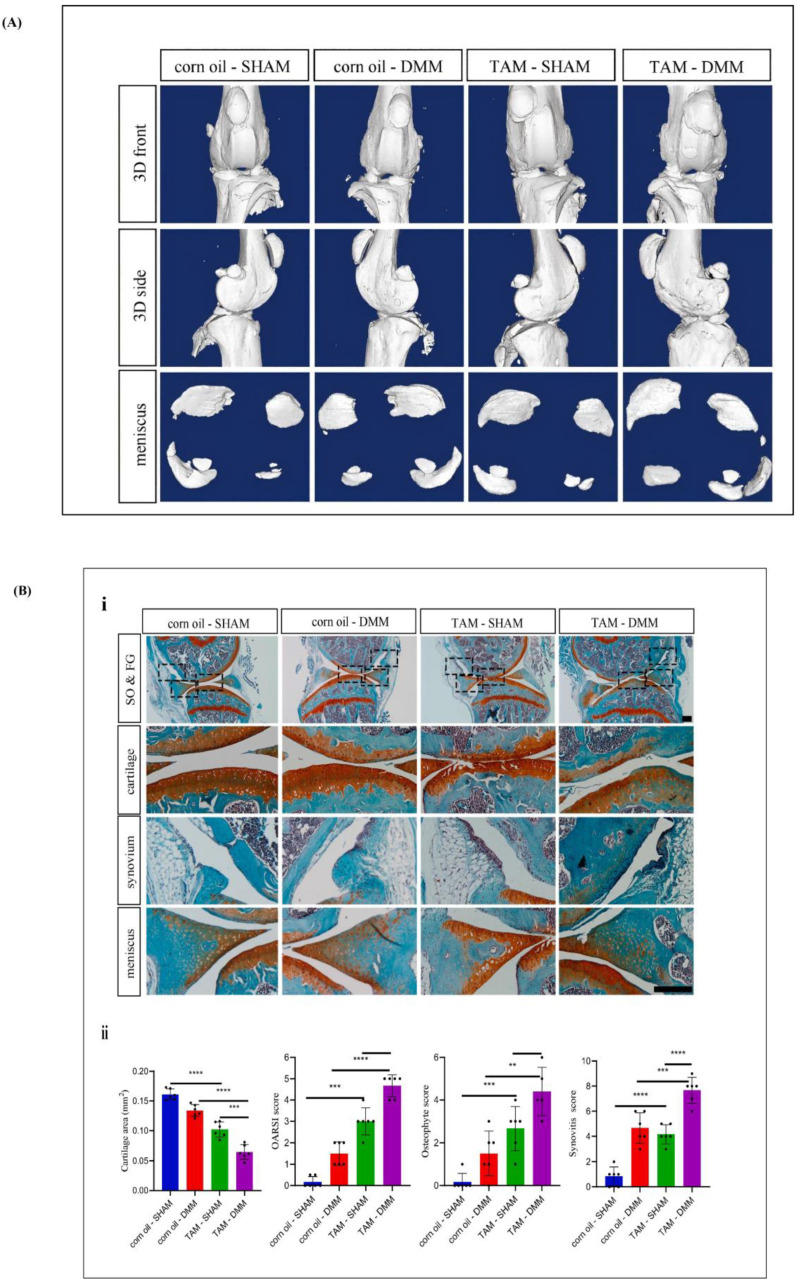
(**A**) μCT of the knee joint sections from DMM-treated Prg4^GFPCreERT2/+^ mice. Reprinted with permission from Ref. [91], copyright 2023, Yao et al. (**B**) (**i**) SO&FG staining on paraffin sectionsof the corn oil and tamoxifen injected Prg4GFPCreERT2/þ; Fermt2fl/fl and tamoxifen injected AggrecanCreERT2/þ; Fermt2fl/fl mice. (**ii**) Quantification of OARSI score; Quantitative analysis of the cartilage area; Osteophyte score evaluated by using Krenn’s synovitis scoring system: Synovitis score was performed using histological sections. Quantitative data are shown as mean s.d. ** *p* < 0.05, *** *p* < 0.001, **** *p* < 0.0001. n ¼ 6 mice per group and results from one representative replicate are shown. Reprinted with permission from Ref. [91], copyright 2023, Yao et al.

The proteins on the cartilage surface are the main boundary lubricants, with lubricin playing a key role in joint boundary lubrication [44]. After proteolysis with trypsin, Chan et al. observed a significant increase in the COF in both the load-bearing and non-load-bearing areas of the joint [87]. Studies have also shown that cartilage from PRG4-deficient mice also shows a higher COF than cartilage from normal mice [88,89]. It was also observed that DMM-induced PRG4-deficient OA mice had higher COF values, suggesting a link between PRG4 and OA (As shown in Figure 3) [90,91]. Neu et al. studied the relationship between the downregulation of SZP and the pathogenesis of OA [92]. When joint motion supports boundary lubrication in surface sliding, SZP acts as a protective barrier, preventing direct solid–solid contact within the joint [23,93].

It is well known that the COF depends on length, size, and operating conditions [67]. In order for SZP to function properly in a boundary lubrication system, the adsorbed molecular films must conform to a closed surface morphology arrangement [68]. A study suggests that SZP may be ineffective in reducing friction under joint boundary lubrication in advanced OA, with other mechanisms dominating the tribological response [94]. In advanced OA, increased surface roughness and tissue stiffness worsen local stress at contact points, leading to the rapid removal of SZP, which cannot be replenished in time [95,96]. This may be due to increased surface roughness in OA samples promoting other friction mechanisms such as collision deformation, adhesion, and plowing [97,98].

Lubricin is a macromolecule found in joint synovial fluid, along with HA and phospholipids, which plays a key role in the boundary lubrication of articular cartilage. It is found on the cartilage surface, where it interacts with HA to form a lubricating layer and reduce friction between articular cartilage surfaces [45,99,100]. Xie et al. observed early articular cartilage changes in OA and explored the therapeutic potential of a bionic brush-like nanofiber for cartilage regeneration. The study found that healthy cartilage contains a brush-like structure with an HA backbone and two key side chains (lubricin and lipid), crucial for joint lubrication. Conjugation of the HA main backbone to lubricin-like polymers or lipid-like polymers stimulated cartilage regeneration in a rat model of early OA [11,101]. In conclusion, SZP lubricants play an important role in boundary lubrication, which no longer functions in severe OA, possibly due to other friction-causing mechanisms that make it difficult to replenish and maintain SZP lubricants. In this case, SZP lubricants cannot play a therapeutic role. Therefore, in the case of boundary lubrication, supplementing SZP may be a means of improving OA.

### 2.4. Phospholipids

Previous studies observed surface phospholipids (PLs) on cartilage and synovial fluid using electron microscopy and mass spectrometry combined with chromatography [102]. In the case of boundary lubrication, there is molecular contact between sliding surfaces [87,103]. PLs with high hydration bead groups exposed to the hydration boundary lipid layer provide effective boundary lubrication, with COFs ranging from 10^−2^ to 10^−4^ during sliding [103,104,105,106]

The synovial fluid of OA patients contains a higher level of PLs, and the concentration of different PLs varies between OA phases. Changes in the PL content may affect joint lubrication [107]. Marta et al. studied the types of PLs in the synovial fluid of normal and OA patients and determined the types of PLs using electrospray ionization tandem mass spectrometry [51]. Gale et al. identified eight different kinds of phosphatidylcholines in human synovial fluid. These are saturated lipid mixtures, including diacylphosphatidylcholine (DLPC), palmitoyloleoylphosphatidylcholine (PLPC), dipalmitoylphosphatidylcholine (DPPC), and palmitoylstearoylphosphatidylcholine (PSPC), as well as unsaturated lipid mixtures, including palmitoyl phosphatidylphosphatidylcholine (POPC), dioleoyl phosphatidylcholine (DOPC), stearoyl oleylphosphatidylcholine (SLPC), and oleoyl stearoyl phosphatidylcholine (OSPC) [10]. DPPC is a saturated amphoteric surfactant found in the synovial joint system, comprising 8–11% of total OSPC, and is used to treat OA [108]. The literature has shown that the articular injection of exogenous dipalmitoic DPPC can reduce WOMAC scores and the Lequesne index by 80–90%, suggesting that the pathogenesis of OA may be reversed [109]. It has been documented that the initial friction level can be determined using specially designed friction devices. When the DPPC concentration is 200 mg/mL, friction is reduced by 46.7%, and the DPPC concentration may be related to the lubrication ability [108].

**Figure 4:**
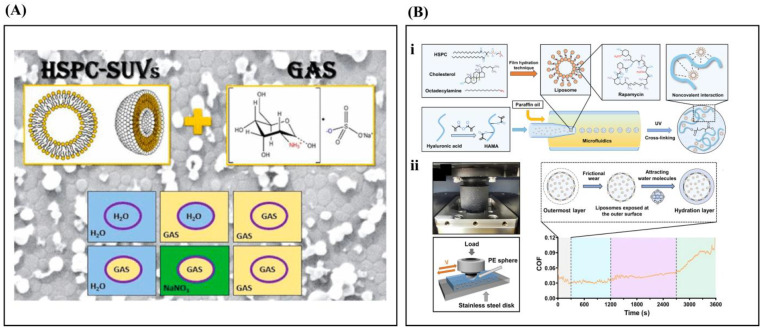
(**A**) Effects of HSPC-SUV (small unilamellar vesicle) and encapsulated glucosamine sulfate (GAS) shear (friction) interactions in aqueous environment. GAS/H_2_O and NaNO_3_/H_2_O are two liposomes composed of aqueous nuclei and suspended in different bulk solutions: gas and sodium salt. Reprinted with permission from Ref. [114], copyright 2014, Klein et al. (**B**) (**i**) RAPA@Lipo@HMs were prepared by combining RAPA@Lipos made of HSPC and cholesterol with photo-crosslinking HAMA matrix and microfluidics. (**ii**) Photograph and schematic of the UMT-3. PE, polyethylene. COF–time curve for the newly prepared Lipo@HMs. Reprinted with permission from Ref. [112], copyright 2022, Lei et al.

In conclusion, PLs play a role in joint lubrication. Different types and concentrations of PLs affect lubrication, and the DPPC concentration influences its performance. More research is needed on the factors affecting the use of PLs as a lubrication supplement [11,110]. DPPC is an exogenous supplement that has been clinically used in the treatment of OA. Researchers have designed many multi-functional lubricant liposomes inspired by the structure and properties of PLs (Figure 4) [111,112,113].

## 3. Multifunctional Lubricant Strategy

Hydrogels, liposomes, NPs, and how their features were verified to affect lubrication are summarized in Table 2. Some of these materials are drug-carrying, while others are cell-carrying, with the ultimate goal of aiding OA recovery.

### 3.1. Hydrogels Loaded with Anti-Inflammatory Drugs

Oral analgesics are used as first-line therapeutic agents [134]. Acetaminophen is preferred for its low cost and safety, along with NSAIDs (such as ketorolac), COX-2 inhibitors (such as diclofenac, ibuprofen, celecoxib, and rofecoxib), and opioids [135]. Chronic oral administration of these drugs can produce a variety of serious side effects, such as gastrointestinal, cardiovascular, renal, and central nervous system complications [136]. Glucocorticoids are another important class of drugs used in the treatment of OA [137].

OA has long been recognized as a cartilage-destroying chronic disease [138]. OA was initially thought to result from increased joint pressure or brittleness of the cartilage matrix [139]. Inflammatory factors from synovitis and subchondral bone degradation contribute significantly to OA [17]. Recent evidence supports OA as an inflammatory process involving metabolic syndrome, innate immunity [140], and low-grade inflammation, which plays a key role in disease progression [104]. Consequently, loading anti-inflammatory drugs into a hydrogel and then injecting it directly into the joint is a promising approach for treating OA. It is hoped that this approach will improve OA treatment [118,119].

**Figure 5:**
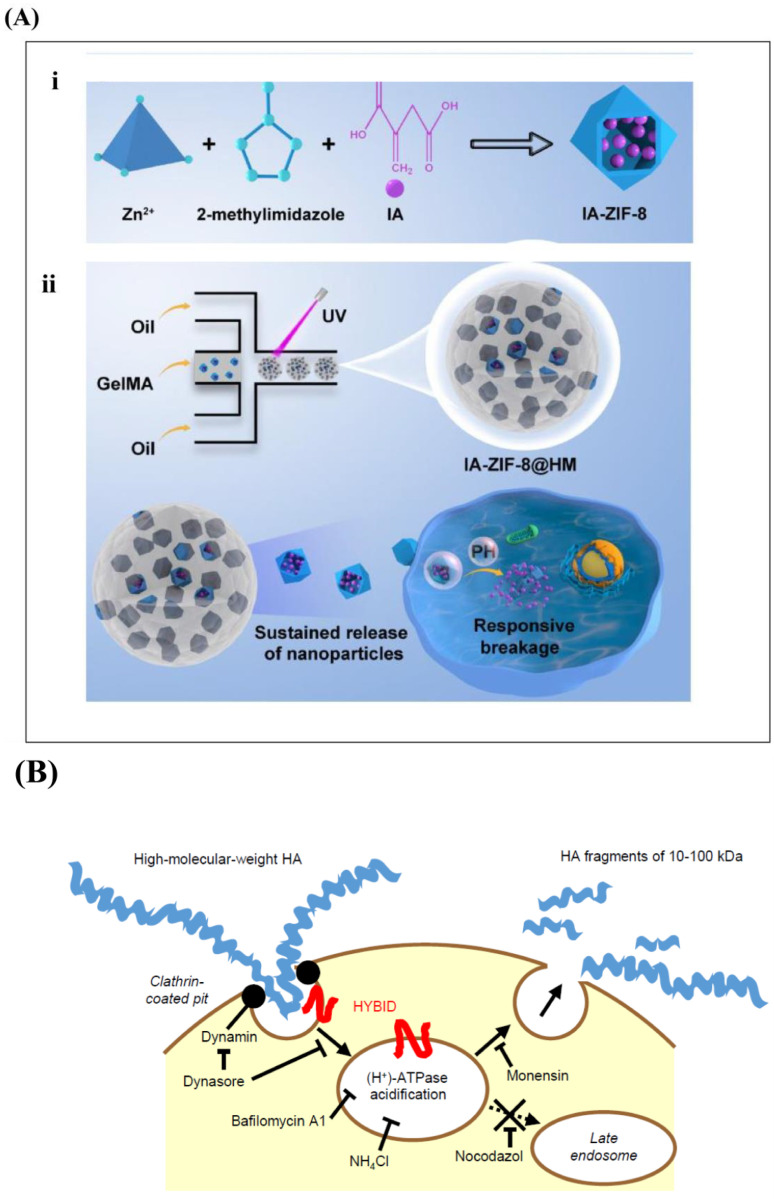

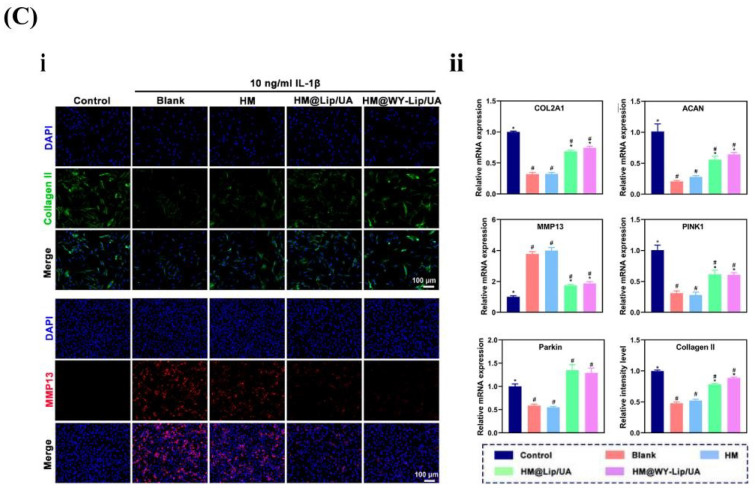
(**A**) (**i**) The synthesis of itaconate (IA)-encapsulated zeolitic imidazolate framework-8 (IA-ZIF-8) nanoparticles. (**ii**) The fabrication of IA-ZIF-8-loaded hydrogel microspheres (IA-ZIF-8@HMs) via one-step microfluidic technology under ultraviolet (UV) light and the design of IA-ZIF-8@HMs for treating OA. Reprinted with permission from Ref. [142], copyright 2023, Yu et al. (**B**) The construction of microfluidic hydrogel microspheres (HM@WY-Lip/UA) and the injection of HM@WY-Lip/UA for the treatment of OA by activating PINK1-Parkin-mediated mitophagy. Reprinted with permission from Ref. [119], copyright 2024, Chen et al. (**C**) (**i**) Cellular homeostasis maintenance. Immunofluorescence analysis of Collagen II and immunofluorescence analysis of MMP13. (**ii**) mRNA expression of COL2A1, ACAN, MMP13, PINK1 and Parkin. n = 3 for each group; # and * indicate statistical significance (*p* < 0.05) when comparing with the control and blank groups, respectively. Reprinted with permission from Ref. [119], copyright 2024, Chen et al.

It has been reported that the IA injection of diclofenac acetaldehyde HA (SI-613) exerted an effective and long-lasting analgesic effect in experimental OA models. HA has lubricating and anti-inflammatory properties by itself, and it can also relieve pain after loading with a double anti-inflammatory drug, making it a promising therapeutic modality [141]. Yu et al. designed IA-ZIF-8@HMs for treating OA, which exhibited both pH responsiveness and protonated acid responsiveness. IA can regulate joint inflammation and intracellular oxidative stress (as shown in Figure 5A) [142]. Hanafy et al. developed a hydrogel with Porroxam 407 (PX) as a gelling agent and HA/DK as an anti-inflammatory, promoting cartilage regeneration in a mouse OA model [143]. Jin et al. developed an HA/gelatin composite hydrogel with EGCG for a surgically induced OA model. Histologic analysis and inflammatory testing showed effective inflammation control and cartilage regeneration [140]. Researchers designed a PL407-PL338-HA-Sulforaphane (SFN) hydrogel. SFN has anti-arthritic and immunomodulatory activity and reduces metalloproteinase expression by downregulating the NF-κB pathway, preventing cartilage degeneration in OA [144]. Lei et al. constructed a microfluidic hydrogel microsphere (HM@WY-Lip/UA) and injected it directly into the joint cavity to treat OA by activating pink1-parkin-mediated mitochondrial autophagy [134] (as shown in Figure 5B).

**Figure 6:**
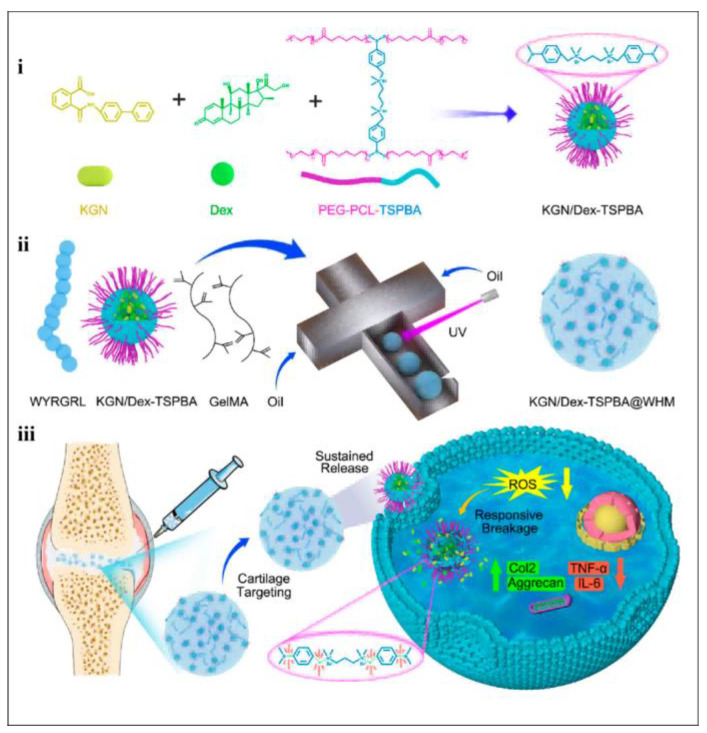
(**i**) The fabrication of kartogenin (KGN) and KGN/dexamethasone (Dex)-TSPBA nanoparticles. (**ii**) The fabrication of KGN/Dexamethasone (Dex)-Tetra-n-Propyl Ammonium Bromide (TSPBA)@WYRGRL hydrogel microspheres (WHMs). (**iii**) The mechanism of KGN/Dex-TSPBA@WHMs in the treatment of OA. Reprinted with permission from Ref. [145], copyright 2022, Yu et al.

Drug-loaded hydrogel microspheres have many shortcomings. Other ros-responsive hydrogels have since been prepared to improve therapeutic efficacy in OA. Yu et al. developed injectable HMS KGN/Dex-TSPBA@ WHMs. The phenylboronic acid bond (PBA) in MS releases the drug in response to ROS, the KGN promotes the differentiation of stem cells towards cartilage, while the Dex alleviates inflammation and reduces cartilage degradation. A WYRGRL-targeting peptide enhances the ability of nanoparticles to target cartilage, allowing therapeutic agents to accurately treat cartilage [145] (as shown in Figure 6). In conclusion, injecting hydrogels with anti-inflammatory drugs into the OA joint cavity not only reduces inflammation but also aids in lubrication recovery and cartilage formation, promoting better OA rehabilitation.

### 3.2. Stem Cell-Loaded Lubricating Hydrogels

**Figure 7:**
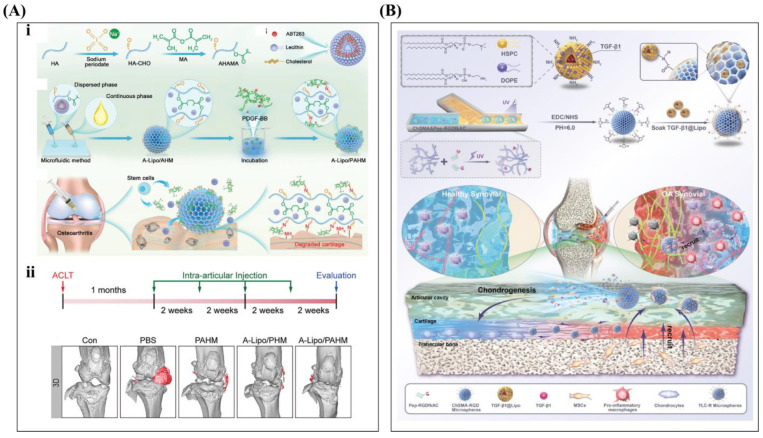
(**A**) (**i**) Design and preparation of A-Lipo/PAHM and A-Lipo; synthesis of AHAMA hydrogels via sodium periodate oxidation and MA modification; treatment of OA with intra-articular injection of A-Lipo/PAHM. Degraded cartilage in OA exposes numerous amino groups, and aldehyde-modified A-Lipo/PAHM localizes to degraded cartilage through Schiff base reaction. Released PDGF-BB recruits endogenous stem cells to repair damaged cartilage. Reprinted with permission from Ref. [121], copyright 2024, Xiong et al. (**ii**) Gait analysis and micro-CT to evaluate OA treatment effect in vivo. Reprinted with permission from Ref. [121], copyright 2024, Xiong et al. (**B**) Scheme of TLC-R production and its application in OA treatment. TGF-β1@Lipo was prepared via membrane dispersion method. Microfluidic device was used to collect continuous droplets, and UV crosslinking was performed. ChSMA-RGD porous microspheres were formed. TLC-R can treat OA by recruiting pro-inflammatory macrophages and MSCs, reducing inflammation, promoting chondrogenic differentiation, modulating cartilage metabolism, and lubricating joints. Reprinted with permission from Ref. [33], copyright 2024, Zhou et al.

Stem cell therapy is a method of treating or preventing disease by utilizing the regeneration and differentiation potential of stem cells. Stem cells are a class of undifferentiated or hypo-differentiated cells that have the ability to self-renew (self-replicate) and differentiate into a variety of cell types. Cellular components, carrier or matrix scaffolds, and bioactive components are the three core elements of tissue engineering. During the differentiation of MSCs into cartilage, the cells are influenced by growth factors and regulated by the three-dimensional (3D) environment they reside in [136,146]. A 3D scaffold is crucial for in situ stem cell transplantation, as it immobilizes the stem cells, promotes proliferation, and protects them from unfavorable environmental factors [147]. Xiong et al. prepared an AHAMA hydrogel with high sodium oxidation and MA modification and loaded it with pro-apoptotic liposomes (Navitoclax (ABT263) encapsulated, A-Lipo) and PDGF-BB. A-Lipo induces apoptosis in senescent chondrocytes (SN-chondrocytes), which are engulfed by macrophages, remodeling endocytosis to protect normal chondrocytes and maintain MSC chondrogenic differentiation (Figure 7A). Li et al. found that the intra-articular injection of CD146+ adipose-derived mesenchymal stem cells (ADSCs) had better inflammation-modulating effects in a rat osteochondral defect model. Further experiments demonstrated that combining CD146 + ADSCs with articular cartilage extracellular matrix (ACECM) scaffolds reduced subcutaneous inflammation and promoted better cartilage regeneration over time [146]. Stem cell-loaded hydrogel scaffolds combined with liposomes carrying anti-inflammatory drugs effectively reduce inflammation and promote stem cell differentiation and cartilage regeneration, offering a promising approach to OA treatment [33,121].

BMSCs are considered an important source of seed cells in cartilage tissue engineering [137,148,149]. Zhang et al. injected an HA/collagen hydrogel system containing stem cells and TGF-β1 into injured cartilage in rats, promoting cartilage regeneration, reconstruction, and lubrication recovery [147]. Liu et al. designed UCMSCs loaded with a graphene oxide (GO) particle lubricant, promoting chondrocyte secretion, reducing joint inflammation, and aiding cartilage repair, offering a potential OA treatment [150]. Wang et al. added icariin (ICA) and BMCs to a self-assembled peptide nanofiber hydrogel scaffold to promote the differentiation of bone marrow mesenchymal stem cells into chondrocytes. In the DMM-induced OA model, the OARSI and Mankin scores of the hydrogels containing BMSCs and ICA were significantly lower compared to the cell and icariin groups. These results suggest that it may be a treatment for OA [151]. Zhou et al. used microfluidic and chemical crosslinking technology to prepare microspheres containing TGF-β1 liposomes (TLC-R). TLC-R can reduce inflammation, promote the chondrogenic differentiation of BMSCs, and regulate OA metabolism by recruiting pro-inflammatory macrophages and mesenchymal stem cells (as shown in Figure 7B). The intra-articular injection of BMSCs directly supplements chondrocyte numbers through stem cell differentiation, promoting lubrication recovery and aiding OA rehabilitation. Although there are many uncontrollable factors in the conversion process, it can be considered a promising treatment for OA.

### 3.3. Lubricated Drug-Carrying Nanoparticles

Nanoparticles (NPs) have become a hotspot in lubrication research because of their unique mechanical and tribological properties [151]. Based on the mechanical engineering approach of adding nanoparticles to lubricating oil to improve performance, nano-additives were added to a bionic joint lubricant developed for enhanced lubrication [152]. Due to the limitations of the disease, doctors often inject the drug directly into the joint cavity to minimize potential side effects from systemic exposure [153]. However, drugs administered via intra-articular injection are often cleared quickly from the joint space through small veins and lymphatic vessels located on the synovial membrane [154]. NPs can be used to improve lubrication performance. Nanoparticles can establish a local drug reservoir at the target site, effectively addressing the issue of rapid drug clearance and frequent administration [155,156]. Nanoparticles penetrate tiny gaps, altering the tribology of the contact surface, forming a protective layer and acting as ball bearings to enhance lubrication [157]. Lawson et al. added tantalum oxide NPs to bovine synovial fluid to improve boundary lubrication by reducing the COF between cartilage joint surfaces [158]. Lumin et al. synthesized a novel bionic lubricant (CS-NPs). It achieved an extremely low COF (0.01) in friction experiments, has good water lubrication properties, reduces wear, and is expected to be a treatment for OA [159].

**Figure 8:**
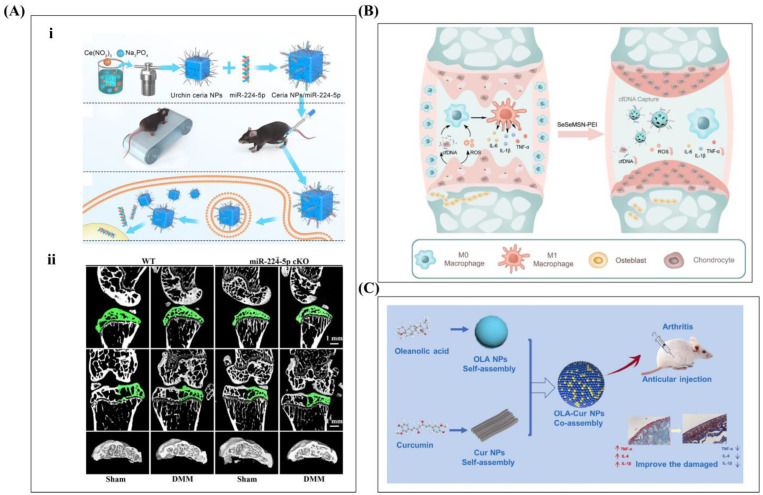
(**A)** (**i**) Ceria NPs were synthesized via a hydrothermal reaction (Ce(NO_3_)_3_·6H_2_O, Na_3_PO_4_, and deionized water) and combined with miR-224-5p. DMM mice underwent high-intensity treadmill training to induce OA, followed by the injection of Ceria NPs/miR-224-5p into the knee joint. These particles were adsorbed onto the cell membrane, underwent endocytosis, and released miR-224-5p. Reprinted with permission from Ref. [128], copyright 2023, Chen et al. (**ii**) The representative 3D images, sagittal and coronal two-dimensional images and subchondral bone three-dimensional images of the knee joint of chondrocyte specific mir-224-5p deletion mice, were reconstructed using micro-CT. Reprinted with permission from Ref. [128], copyright 2023, Chen et al. (**B**) Design schematic of MSN-PEIs with dual-targeting cfDNA and ROS bridged by diselenide ether for treating OA by modulating damage and inflammation. Reprinted with permission from Ref. [133], copyright 2023, Shi et al. (**C**) Schematic illustration of OLA-Cur NPs for anti-inflammatory and antioxidant treatment of OA. Reprinted with permission from Ref. [129], copyright 2025, Liu et al.

Kai et al. added liposome NPs to an HA solution, and tribological tests showed that they could improve the anti-friction properties of the HA solution and significantly reduce its COF [160,161]. Kang et al. believe that self-assembled HA-NPs resist hyaluronidase digestion and remain in joint cavities for extended periods, enhancing lubrication performance [162]. Zheng et al. synthesized HA-based zwitterionic nanospheres and grafted 2-methylacryloxyethylcholine phosphate (MPC) onto HA to form HA-MPC nanospheres. The phosphocholine groups provide excellent lubrication via hydration [162]. Friction experiment results showed that HA-MPC nanospheres improved lubrication under shear force, and the friction coefficient was reduced by 40% compared with HA [127]. In addition, multifunctional NPs were designed [123,130]. Liu et al. used a supramolecular co-assembly strategy to construct an oleanol–curcumin (OLA-Cur) co-assembled composite nanosustained release processing system. The OLA-Cur NPs inhibited the release of the pro-inflammatory cytokines TNF-α, IL-6, and IL-1β from LPS-induced RAW 264.7 macrophages, promoted IL-10 secretion, and improved oxidative stress in hydrogen peroxide-induced synovioblasts [163] (as shown in Figure 8A).

Chen et al. identified microRNA-224-5p (miR-224-5p) from OA patient samples and noted that it protects cartilage from degeneration. They synthesized sea urchin-like ceramic nanoparticles to enhance gene therapy for OA (as shown in Figure 8C). Sheng et al. designed reactive oxygen species (ROS) sensitive Fenofibrate (FN)-loaded nanoparticles (FN-CNPs). The FN-CNPs may alleviate OA by inhibiting chondrocyte ferroptosis [123], which has become a key target of OA therapy. The role of phospholipid-coated mesoporous silica nanoparticles (MSPs) was investigated by Tao et al. PLs have an excellent hydration lubrication mechanism. A series of tribological experiments were conducted under different experimental conditions, and the results showed that MSN@lip significantly reduces the COF compared to non-PLS-coated silica. This lubricating nanocarrier may represent a promising strategy [164]. Shi et al. prepared polyethyleneimine (PEI) functionalized diselenide-bridged mesoporous silica nanoparticles (MSN-PEIs) with cell-free DNA (cfDNA) binding and antioxidant properties. Mechanistically, the multi-target blockade mitigated oxidative stress and inhibited cfDNA-induced inflammation by inhibiting macrophage M1 polarization (as shown in Figure 8B). Li et al. used 2-methacryloyloxyethyl phosphate (PMPC) as a modifier to prepare biodegradable mesoporous silica nanoparticles (bMSNs) and prepared lubricating drug-loaded nanoparticles (bMSNs-NH_2_@PMPC) via photopolymerization. The COF of the bMSNs-NH_2_@PMPC was reduced by 50% compared with bMSNs. This is attributed to the hydration of the PMPC polyelectrolyte polymer, which forms a hard hydration layer around the amphiphilic ionic head group (N^+^(CH_3_)_3_ and PO4^−^) layers. This may be a promising approach for the treatment of OA. An increasing variety of drug delivery and lubrication-enhancing nanoparticles have been developed to treat OA [128,129,133].

## 4. Summary and Prospects

OA is a disease characterized by the degeneration of cartilage in the joints, leading to pain and eventually disability. Decreased joint lubrication is a pivotal factor in the initiation and progression of OA [40]. Effective and well-tolerated lubrication strategies are urgently needed for OA treatment [22]. This study reviews the latest advancements in lubricants for OA treatment, including single-function lubricants and multifunctional strategies. Single-function lubricants, which are found on cartilage surfaces and in synovial fluid, effectively reduce friction and wear between joint surfaces, providing potential solutions and new insights for designing synthetic lubricants [1]. Inspired by single-function lubricants in joints, multifunctional strategies involving lubricating hydrogels have been studied; these strategies also incorporate anti-inflammatory drugs, stem cells, and nanoparticles [136,165] and not only provide lubrication but also alleviate inflammation and may promote stem cell differentiation into chondrocytes, offering a more effective approach to treating OA treatment. Although these lubricants exhibit good lubrication properties, their lubrication and physiological mechanisms in vivo remain unclear [2]. More importantly, these lubricants degrade in synovial fluid, presenting another challenge [166]. Future research should focus on in vivo studies and clinical applications to better understand lubrication mechanisms and address practical clinical issues while designing multifunctional treatments with holistic benefits [167]. This could offer opportunities to overcome degradation issues and achieve longer-lasting OA treatments. We believe that the lubrication strategies reviewed here may directly contribute to and lay the foundation for future OA treatments.

## Figures and Tables

**Table 1:** Summary of modified substances and friction tests for single-function lubricants.

Materials	Treatment Method	Friction Pairs	COF	Measure Equipment	Ref.
APTES with chemically grafted HA	DOPC	Mica	≈0.50	SFA	[40]
Bovine articular cartilage	Enzymatic treatment and PBS	Glass	0.12 ± 0.03	TRB^3^	[41]
CS	Chondroitinase ABC	Glass	3.10 ± 0.04	Reciprocating motion pin-on-plate machine	[42]
Lubricin	NA	Polished glass	0.90 ± 0.011	Tribometer	[43]
SZP, HA, SAPL	Enzyme	Triangular Si_3_N_4_	0.23 ± 0.03	AFM	[44]
SZP in patients with OA	PBS	Glass disk	NA	Tribometer	[45]
HA/DPPC	NA	Mica surfaces	≈0.001	SFB	[10]
Ovine cartilage	PBS	Silicon	0.43	AFM	[46]
HA and hylan	PBS	Bilayer mica surface	0.15–0.27	SFA	[22]
Sliding cartilage surfaces	NA	Glass	0.001	AFM	[37]
Bovine synovial fluid	Saline	Glass	0.012–0.015	Tribometer	[47]
SZP in cattle joint	Saline	Glass	NA	AFM	[48]
Lubricin mimic	Trypsin	Silicon	NA	AFM	[49]
Mouse cartilage	Saline	Silicon	NA	AFM	[50]
HA + PLs	PBS	Mica surfaces	0.02–0.03	AFM	[51]
HSPC, DMPC, POPC	NA	Silicon tip	0.001	AFM	[50]
DMPC, DPPC, DSPC	NA	Mica surfaces	0.0001	SFB	[52]

Note: The COF of a normal joint ranges from 0.01 to 0.03. Under high physiological pressure, the cartilage surface COF can drop to as low as 0.001 [1,53]. These data show that the COF of normal joints is very low, which contributes to the flexible movement of joints and reduces wear on joint surfaces. Abbreviations: atomic force microscopy (AFM); surface forces apparatus (SFA); surface force balance (SFB); pin-on-disk tribometer (TRB^3^); amino-propyl-triethoxy-silane (APTES); dioleoyl phosphatidylcholine (DOPC); superficial zonulin proteins (SZP); phosphate buffer solution (PBS); surface-active phospholipids (SAPL); Dipalmitoylphosphatidylcholine (DPPC); phosphatidylcholine (HSPC); Dimyristoylphosphatidylcholine (DMPC); Palmitoyl phosphatidylphosphatidylcholine (POPC); 1;2-distearoyl-sn-glycero-3-phosphocholine (DSPC); Silicon nitride (Si_3_N_4_); not available (NA).

**Table 2:** Summary of modified products and effects for multifunctional lubricant strategy.

Materials	Lubricating Carrier	Cell/Animal Types	Drug Loading	Effects	Ref.
CLX@Lipo@HA-gel	Lipo@HA-gel	C-28/I2 chondrocyte cell/(ACLT + MMx)	CLX	Shear-responsive boundary lubricants and drug-delivery vehicles to alleviate friction-related diseases like OA.	[111]
RAPA@Lipo@HMs	Lipo@HMs	C-28/I2/ACLT	RAPA	Providing efficient lubrication and potentially alleviating friction-related OA.	[112]
LQ@ChsMA@Lipo	ChsMA@Lipo	RAW 264.7/DMM	LQ	Providing a degradable and dual antioxidant drug delivery platform for the treatment of OA.	[34]
AST@Lip-FA	Lip-FA	RAW 264.7/BMDMs/mPCs/ACLT	AST	Providing a biodegradable dual antioxidant drug delivery platform for the treatment of OA.	[115]
TA-NM@Lip	NM@Lip	RAW 264.7/ACLT + MMx	TA	Representing a promising nanotherapeutic approach for OA therapy.	[116]
MLX-Ca(AC)_2_Lipo	Ca(AC_)2_Lipo	ATDC5	MLX	Nanodrugs with dual anti-inflammatory and lubricating functions for the treatment of OA.	[117]
FCM@Lipos-RSG	Lipos-RSG	Chondrocytes/DMM	RSG	Advancing orthopedic treatments, particularly OA, by enhancing specific targeting and multifunctionality.	[113]
KGN-loaded GelMA@Lipo microgels	GelMA@Lipo microgels	BMSCs/DMM	KGN	Reducing osteophyte burden and preventing articular cartilage degeneration as well as subchondral bone changes when intra-articular injection in a surgically induced rat OA model.	[118]
HM@WY-Lip/UA hydrogel microspheres	HM@WY-Lip	Chondrocytes/DMM	UA	Providing a protective effect on cartilage degeneration using hydrogel microspheres with mitochondrial orientation.	[119]
ChsMA + CLX@Lipo@GelMA hydrogel microsphere	ChsMA + Lipo@GelMA	Chondrocytes/ACLT	CLX	Demonstrating a beneficial impact of the outer shell in reducing inflammation. While the inner methacryloyl microsphere core degraded, chondroitin sulfate is released to promote chondrocyte anabolism and facilitate cartilage repair.	[120]
HAMA/MMP13sp/Lipo@celecoxib microspheres	HAMA/MMP13sp/Lipo	C-28/I2/ACLT	CLX	Demonstrating specific enzyme responsiveness for precise anti-inflammatory drug release. The MMP13-responsive hydrogel microsphere system achieves intelligent and controllable drug release in OA.	[31]
A-Lipo/PAHM	A-Lipo/PAHM	Chondrocytes + BMSCs/ACLT	ABT263	Confirming that hydrogel microspheres localized to cartilage lesion reversed cartilage senescence and promoted cartilage repair in OA.	[121]
TGF-β1@Lipo@ChSMA-RGD microsphere (TLC-R)	Lipo@ChSMA-RG	Chondrocytes + BMSCs/ACLT	TGF-β1	Releasing ChS to further promote chondrocyte synthetic metabolism and inhibit the degradation metabolism and inflammation over a long period.	[33]
AST@Lip-FA nanoparticle	Artesunate DSPC Cholesterol MDSPE-PEG2000	mPCs + RAW 264.7/ACLT	AST	Precisely enriching the inflamed joints, achieving long-term retention, and fully utilizing the anti-inflammatory, antioxidant, and cartilage protective effects of AST to effectively alleviate the progression of OA.	[115]
HA–NSc NPs	HSPC, DOPE, cholesterol, octadecylamine	RAW 264.7/MIA	o-PD	Acting as a dual-action therapeutic agent for the treatment of OA by alleviating pain, inflammation, and joint damage	[122]
FN-CNPs	DSPE-PEG-WYRGRL Dextran-g-PMEMA Fenofibrate	ATDC5/DMM	Fenofibrate	Highlighting the efficacy of FN-CNPs in mitigating OA progression by suppressing chondrocyte ferroptosis via regulating ROS levels, antioxidant systems, and the lipid metabolism of chondrocytes.	[123]
KGN@PLGA/PDA-PEG-E7 NPs	PLGA/PDA-PEG-E7	BMSCs/ACLT	KGN	Inducing cartilage in vitro and protecting the cartilage and subchondral bone in a rat ACLT model.	[124]
C6@BRJ + IgG/C6@BRJ NPs	BR + JPH203	RAW 264.7/ACLT	IgG/BRJ	Involving M1 macrophages to engulf carrier-free BR/JPH203 nanoparticles to suppress inflammation for OA therapy.	[125]
CHP-KGN-An particle	An albumin nanoparticle	Chondrocytes/DMM	KGN	Attenuating cartilage degradation, synovitis, osteophyte formation, and subchondral bone sclerosis in OA model mice and exhibiting a more prominent effect on physical activity improvement and pain alleviation.	[126]
Atb@NP@Raw@CD34	NP@Raw@CD34	RAW 264.7 + HUVECs/DMM	Atb	Attenuating joint degeneration by inhibiting synovium angiogenesis-mediated synovitis. Local injection of Atb@NP@Raw@CD34 presents a promising approach for clinically impeding OA progression.	[127]
miR-224-5p@ceria NPs	Ceria Nanoparticles	FLSs + Chondrocytes + HUVECs/DMM	miR-224-5p	Demonstrating excellent scavenging performance for reactive oxygen species (ROS), which can regulate the microenvironment of OA to further improve the gene treatment of OA.	[128]
OLA-Cur NPs	PVA/Nanoparticles	RAW 264.7/L-cysteine activated papain solution	OLA-Cur	Improving the oxidative stress index of hydrogen peroxide-induced human rheumatoid arthritis synovial fibroblasts and ameliorating cartilage and subchondral bone damage in mouse OA models.	[129]
DEX@Arg-Mn-MPDA	MPDA nanoparticles +Arg-Mn-MPDA (AMM NPs)	RAW 264.7 + ATDC5 + mPCs/ACLT	DEX	Contributing to the prevention of chondrocyte apoptosis through the inflammatory factor-dependent TLR-3/NF-κB signaling pathway. DAMM NPs played a dominant role in scavenging ROS generated in chondrocytes, and DEX-loaded DAMM NPs significantly attenuated the development of OA in a mouse model.	[130]
RSV-loaded PLGA NPs	PLGA NPs	Chondrocytes/DMM	RSV	Alleviating cartilage destruction and improving OA symptoms by downregulating and inhibiting apoptosis and promoting autophagy.	[131]
RES- and cell ROX-loaded ZIF-8 NPs	mPEG-TK + ZIF-8	RAW 264.7/ACLT	RES	As fluorescence detection and ROS regulation therapy, providing new paths for OA diagnosis and treatment.	[132]
Mesoporous silica nanoparticles (MSN-PEI) with cfDNA	MSN-PEI	RAW 264.7 + mPCs/CIOA + DMM	cfDNA	Alleviating oxidative stress and dampening cfDNA-induced inflammation by suppressing the M1 polarization of macrophages. This study suggests a beneficial direction for targeting multiple danger mediators in the treatment of OA.	[133]

Abbreviations: anterior cruciate ligament amputation and medial meniscectomy (ACLT + MMx); Celecoxib (CLX); Rapamycin (RAPA); Liquiritin (LQ); bone marrow-derived macrophages (BMDMs); mouse primary chondrocytes (mPCs); Astaxanthin (AST); Triamcinolone acetonide (TA); medial meniscus destabilization (DMM); Meloxicam (MLX); Rosiglitazone (RSG); bone marrow mesenchymal stem cells (BMSCs); Kartogenin (KGN); UrolithinA (UA); o-Phenylenediamine (o-PD); Immunoglobulin G-conjugated bilirubin/JPH203 (IgG/BRJ); Axitinib (Atb); fibroblast-like Synoviocytes (FLSs); oleanolic acid–curcumin (OLA-Cur); mesoporous polydopamine (MPDA); Dexamethasone (DEX); Zeolite-based imidazolium salt framework-8 (ZIF-8); Resveratrol (RES) cell-free DNA (cfDNA); Collagenase-induced OA (CIOA); Polyvinyl Alcohol (PVA); D; L-lactide-coglycolide acid (PLGA); Resveratrol (RSV) or (RES).
